# A Study to Compare the Analgesic Efficacy of Intrathecal Bupivacaine Alone with Intrathecal Bupivacaine Midazolam Combination in Patients Undergoing Elective Infraumbilical Surgery

**DOI:** 10.1155/2013/567134

**Published:** 2013-05-15

**Authors:** Anirban Chattopadhyay, Souvik Maitra, Suvadeep Sen, Sulagna Bhattacharjee, Amitava Layek, Sugata Pal, Kakali Ghosh

**Affiliations:** ^1^Lilavati Hospital & Research Center, Mumbai 400050, India; ^2^Department of Anaesthesiology and Intensive Care, AIIMS, New Delhi 110029, India; ^3^Department of Anaesthesiology, IPGME&R and SSKM Hospital, Kolkata 700020, India; ^4^Department of Cardiac Anaesthesiology, IPGME&R and SSKM Hospital, Kolkata 700020, India

## Abstract

Spinal anaesthesia, which is one of the techniques for infraumbilical surgeries, is most commonly criticized for limited duration of postoperative analgesia. Several adjuvants have been tried along with local anesthetic for prolonging the duration of analgesia. In this study, we have observed the effect of midazolam as an adjuvant in patients undergoing infraumbilical surgery. In this prospective, randomized, double blinded, and parallel group and open label study of 90 adult patients aged 18–60 years, of American Society of Anaesthesiologists (ASA) status I and II, scheduled for elective infraumbilical surgery, were randomly allocated in two groups. Each patient in group “B” received hyperbaric bupivacaine 12.5 mg along with 0.4 mL of normal saline in the subarachnoid block, and patients of group “BM” received 12.5 mg hyperbaric bupivacaine along with preservative free midazolam 0.4 mL (2 mg). We found that use of midazolam as adjuvant with the local anesthetic in spinal anaesthesia significantly increases the duration of analgesia (median 320 min versus 220 min) and motor block (median 255 min versus 195 min) but decreases the incidence of postoperative nausea-vomiting (PONV).

## 1. Introduction

Spinal subarachnoid block is one of the most versatile regional anesthesia techniques available today. Regional anesthesia offers several advantages over general anesthesia—blunts stress response to surgery, decreases intraoperative blood loss, lowers the incidence of postoperative thromboembolic events, and provides analgesia in early postoperative period. Subarachnoid block provides adequate anesthesia for patients undergoing infraumbilical surgery.

Among the local anesthetics, 0.5% hyperbaric bupivacaine is the most commonly used drug for spinal anesthesia [1]. The most important disadvantage of single injection SAB is the limited duration. Adjuvants have long been used along with local anesthetics to prolong the duration of anesthesia and analgesia. Prolongation of pain relief by various adjuvants like opioids (like morphine [2], fentanyl [3]), ketamine [4], clonidine [5], and neostigmine [6] were investigated by various investigators. However, each drug has its limitations and side effects, and the need for an alternative methods and drugs always exist.

Discovery of benzodiazepine receptors in spinal cord in 1977 [7] triggered the use of intrathecal midazolam for prolongation of spinal anesthesia. In vitro autoradiography has shown that there is a high density of benzodiazepine (GABA-A) receptors in Lamina II of the dorsal horn in the human spinal cord, suggesting a possible role in pain modulation [8].

So far different animal studies have revealed no damage to the spinal cord, nerve roots, or meninges and in vitro studies suggested that clinically useful doses of intrathecal midazolam are unlikely to be neurotoxic [9–12].

## 2. Aims and Objective

In this study, intrathecal analgesia with 0.5% hyperbaric bupivacaine 2.5 mL with 0.4 mL 0.9% normal saline has been compared with 0.5% hyperbaric bupivacaine 2.5 mL plus 0.4 mL (2 mg) preservative free midazolam in a predetermined dose, and two groups will be compared in terms of duration of effective analgesia by time interval between the onset of intrathecal block to time for request for first rescue analgesia and by VAS pain score. Perioperative sedation, hemodynamic changes, peak height of block, time for regression of motor block to sacral dermatome, and any obvious side effect were also assessed.

## 3. Specific Objectives of This Study

1. To compare duration of effective analgesia between the two groups.
2. To assess the perioperative hemodynamic changes.
3. To observe perioperative sedation and any obvious adverse effects.

## 4. Methodology

After obtaining institutional ethics committee clearance and written informed consent from the patients, 90 adult patients of ASA physical status I and II and aged 18–60 years undergoing elective infraumbilical (gynecologic/urologic) under spinal subarachnoid block anesthesia were included into the study. Patients refusing to participate, with known allergic to local anaesthetic and midazolam, suffering from chronic pain, and pregnant women were excluded from the study. Patients having the level of sensory block below T10 after 15 minutes of subarachnoid block or having VAS pain score greater than 40 at any point of time during intraoperative period were offered general anesthesia for the rest of the procedure. This subset of patients were planned to regard as “incomplete block” category and planned to exclude from the final data analysis.

### 4.1. Calculation of Sample Size

Sample size estimation was done using power and sample size calculation software (version 2.1.30, DuPont & Plummer, February 2003). Based on clinical experience and review of the literature an educated guess was made that a 30-minute difference of mean duration of analgesia between two groups would be statistically significant. Within group standard deviation was assumed to be 60. Using this data and assuming a study power of 90% and probability of type I error of 5%, a sample size of 85 patients was found to be required for obtaining statistically significant mean difference of mean duration of analgesia in two groups. So assuming equal distribution of patients in both groups a total number of 90 patients were incorporated in the study (*n* = 90), with 45 patients in each group (*n* = 45).

### 4.2. Randomization and Blinding

A computer-generated randomization table (Microsoft Excel 2007 software) was used to assign each patient into either group “BM” (patients receiving bupivacaine and midazolam during subarachnoid block) or group “B” (patient receiving bupivacaine only). For ensuring blinding, randomly allocated coded syringes of drugs were prepared by an anesthesiologist who did not perform subarachnoid block or record the outcome intraoperative and postoperative period. The investigator and the anaesthesiologist performing the study were blinded to the content of the drugs contained in the syringes. The solution intended for group BM contained 2.5 mL hyperbaric bupivacaine (0.5%) plus 0.4 mL (2 mg) preservative free midazolam, and solution intended for group B contained 2.5 mL hyperbaric bupivacaine (0.5%) plus 0.4 mL normal saline (0.9%). The total volume of subarachnoid injection in either group was 2.9 mL.

### 4.3. Study Protocol

Explanation regarding the procedure and study, education regarding VAS score, and necessary written informed consent were taken during the preoperative checkup ad visit. Standard ASA fasting guidelines were followed in all patients. Patients were not given any premedication.

On arrival at the operation theatre, intravenous access was established with an 18G intravenous cannula in a large vein of forearm, and coloading was done with prewarmed ringer's lactate solution 15 mL/kg body weight, that was infused over 15 minutes.

#### 4.3.1. Procedure of Intrathecal Injection

Under strict aseptic precautions a 25G Quincke spinal needle was introduced into L3-L4 or L4-L5 intervertebral space in midline approach in lateral posture, and, after confirming free flow of CSF, predetermined 2.9 mL of drug solution was injected. The rate of injection was kept 0.2 mL/second. At the end of the injection, a small sterile dressing was applied and patients were placed supine with a pillow under the head and neck soon after administration of intrathecal drugs.

Oxygen (2 L/min) was administered throughout the procedure via nasal cannula. Intraoperative fluid management was done in relation to body weight of the patient, vital signs, and intraoperative losses. At the end of the surgery, the patients were shifted to the postoperative ward for clinical monitoring of vital signs, appropriate fluid therapy, and other treatment.

Assessments of parameters include the following.

- Height of sensory block (by the ability to perceive cold sensation with spirit soaked blunt tipped cotton swab in midaxillary line bilaterally) every 5 minutes for first 30 minutes and then at 15-minute interval till the end of surgery and hourly thereafter till rescue analgesic was required. Duration of sensory analgesia was calculated with two dermatome regression from peak block height for each patient.
- Motor block was assessed using a 6-point-modified Bromage scale [13] (1 = complete motor block; 2 = almost complete blockade, the patient is able to move feet only; 3 = partial motor blockade, the patient is able to move the knees; 4 = detectable weakness of hip flexion, the patient is able to raise the leg but is unable to keep it raised; 5 = no detectable weakness of hip flexion; 6 = no weakness at all). These measurements were performed at 5, 10, and 15 min after intrathecal injection and then every 15 min after surgery until no motor blockade regressed to sacral dermatome. Duration of motor block was calculated till modified Bromage value reached six for each patient.
- Hemodynamic changes—heart rate, blood pressures—every 5 minutes for first 30 minutes and then at 10-minute interval till the end of surgery and hourly there after till rescue analgesic are required. Only hemodynamic changes that require treatment as described below were considered significant.
- Visual analogue scale (VAS) score is a tool for assessment of analgesia in the intraoperative and postoperative period. VAS pain score is a linear pain scoring tool ranging from 0 to 100 mm where patients marked a circle around a point (0, 10, 20, 30, etc.) on a 100 mm scale. Duration of analgesia was defined from the administration of subarachnoid block till patient demanded for rescue analgesia or VAS greater than 40 which ever earlier.
- Level of sedation (assessed by four-point ordinal scale 0 = awake, alert; 1 = drowsy, responds to call; 2 = drowsy, responds to tactile stimulus; 3 = deep sedation, unresponsive) 5 minutes for first 30 minutes and then at 10-minute interval till the end of surgery and hourly there after till rescue analgesic was required.
- Side effects such as nausea and vomiting, pruritus, and respiratory depression were observed in the intraoperative period and in the postoperative period till requirement of rescue analgesic.

Episodes of intraoperative side effects such as hypotension (a decrease in systolic blood pressure of more than 20% from baseline) and bradycardia (HR < 45/min) were recorded. Hypotension was treated with bolus of ringer lactate and incremental doses of mephentermine 6 mg IV, and bradycardia was treated with atropine sulphate 0.6 mg IV bolus.

Rescue analgesia was administered postoperatively when VAS score >40 or when patient requested for analgesia with diclofenac sodium 1 mg/kg body weight intramuscularly. Time from institution of successful subarachnoid block to request for first rescue analgesia was recorded. The study was terminated when patient requested for first rescue analgesia or VAS reached greater than 40 which ever earlier.

### 4.4. Analysis of Data

All raw data of study parameters were entered into a Microsoft excel spread sheet and analyzed using IBM SPSS v17.0. The categorical variables were analyzed using Mantel-Haenszel chi-square test or Fischer exact test as appropriate. Parametrical numerical valuables were analyzed using independent sample *t*-test. All statistical analysis was two tailed, and a *P* value of <0.05 was considered statistically significant.

## 5. Results

The patients in both groups were comparable in terms of demographic profile, that is, age, height, weight, sex, ASA PS distribution, duration of surgery, and types of surgeries (Table 1).

Twenty eight patients in group B and thirty one patients in group BM had at least one episode of hypotension requiring fluid bolus or vasopressor; however, no statistical difference has been found. Three patients in group B and two patients in group BM had one episode of bradycardia and required intravenous atropine injection.

**Figure 1:**
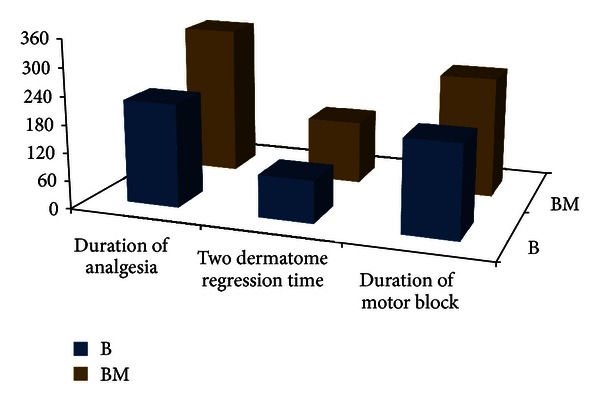
Comparison of median duration of analgesia, two dermatome regression time, and duration of motor block in two groups.

Median peak height of sensory block was up to T4 dermatome in both groups. However, duration of motor block as defined above (median 255 min versus 195 min) and two dermatome regression time of sensory block (median 135 min versus 90 min) have been found to be significantly higher in BM group (Table 2, Figure 1).

Incidence of intraoperative sedation was higher in patients who received midazolam, but the incidence of intraoperative nausea-vomiting and incomplete sensory block (Table 3) was similar between two groups.

**Figure 2:**
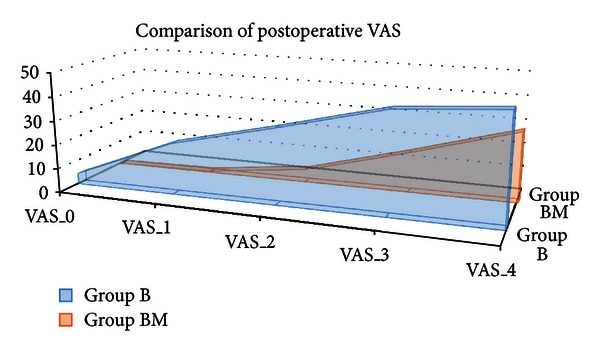
Area under curve of VAS-hr in two groups.

The duration of analgesia was significantly higher in patients receiving bupivacaine and midazolam in comparison to bupivacaine alone (median 320 min versus 220 min) (Figure 1). VAS score was found to be significantly higher (*P* < 0.05) among the patients who received only bupivacaine in 0 (immediately in the postoperative recovery room), 1st, 2nd, 3rd, and 4th hours in the postoperative period. After 4th hour, the comparison was not possible because all patients in group B had received rescue analgesia by that time. Area under curve for the postoperative VAS score-time (mm-hr) is also significantly higher in group B (119.8 versus 37.48) (Figure 2). Incidence of PONV is significantly lower in patients who received intrathecal midazolam; however, sedation score was similar in the postoperative period and none of the patients experienced pruritus or respiratory depression in the postoperative period (Table 4).

## 6. Discussion

The baseline characteristics in either group were similar from statistical standpoint. Considering the intraoperative hemodynamic variables the result of our study is comparable with studies done by Batra et al. [14], Kim and Lee [15], Agrawal et al. [16], and Gupta et al. [17] who also did not find statistically significant difference in heart rate, arterial blood pressure in their studies. Incidence of hypotension and bradycardia is found to be similar in both groups.

The most significant finding of our study is a significant prolongation of sensory blockade which is reflected as the time to request first rescue analgesic and two dermatome regression time of sensory block, both of which were significantly higher in patients receiving midazolam as adjuvant. Batra et al. [14] in 1999 reported a similar finding; they concluded that intrathecal administration of midazolam along with bupivacaine produces better postoperative analgesia and a prolonged sensory blockade; however, they used a much smaller sample size. Kim and Lee [15] in 2001 in a meta-analysis concluded that addition of 1 or 2 mg of intrathecal midazolam prolonged the postoperative analgesic effect of bupivacaine by approximately 2 h and 4.5 h, respectively, compared with controls after hemorrhoidectomy, and this finding suggested a dose-dependent action of intrathecal midazolam. However, we should keep in mind that hemorrhoidectomy pain can be alleviated only by sacral sensory nerves. Our patients were undergoing infraumbilical gynecologic or urologic surgeries, and, for effective analgesia, these patients require blockade of lower thoracic and lumber dermatomes as well. However, we also found that intrathecal midazolam at a dose of 2 mg significantly prolongs the duration of motor block also which may be an important consideration where early ambulation is desirable. Bharti et al. [18] in 2003 reported a prolonged sensory and motor block following midazolam administration as adjuvant with bupivacaine in lower abdominal surgery.

We have found significant difference in sedation level in intraoperative period but not in the postoperative period. Whether intrathecal midazolam causes clinically significant sedation or not is a debatable issue; Yegin et al. [19] found that 2 mg intrathecal midazolam causes significant sedation, but others did not [14, 20]. We think that intraoperative sedation may be a desirable property of intrathecal midazolam.

We also have found that there is a decrease in the incidence of PONV in patients who received intrathecal midazolam. Prakash et al. [21] also reported similar findings in patients undergoing cesarean section. However, it should be interpreted with caution as none of the studies have adequate power to detect a difference in the incidence of PONV.

## 7. Conclusion

We conclude that the addition of 2 mg preservative free midazolam to 0.5% hyperbaric bupivacaine for subarachnoid block in infraumbilical surgery prolongs the duration of effective analgesia as compared to bupivacaine alone and delays the need for postoperative rescue analgesics without having any sedative effect, pruritus, or respiratory depression. The use of intrathecal midazolam also decreases the incidence of postoperative nausea-vomiting (PONV). Intrathecal midazolam in a dose of 2 mg does not have any clinically significant effect on perioperative hemodynamics.

## Figures and Tables

**Table 1:** Demographic profiles of the patients in two groups.

	Group B (*n* = 45)	Group BM (*n* = 45)	Significance
Age (year)	46.56 ± 12.63	42.71 ± 11.75	NS
Height (cm)	160.71 ± 5.59	161.89 ± 6.25	NS
Weight (kg)	58.37 ± 5.95	58.91 ± 6.25	NS
Sex (male/female)	30/15	31/14	NS
ASA PS (I/II)	24/21	25/20	NS
Duration of surgery (min)	79.78 ± 31.5	83.22 ± 31.10	NS
Types of surgery (myomectomy/vaginal hysterectomy/TURP/ TURBT)	8/7/15/15	7/7/16/15	*P* = 0.99^#^

^#^Fisher exact probability test.

TURP: transurethral resection of prostate, TURBT: transurethral resection of bladder tumor.

**Table 2:** Characteristics of subarachnoid block in patients of two groups.

	Group B (*n* = 45)	Group BM (*n* = 45)	Significance
Peak height of sensory block	T4	T4	NS
Two dermatome regression time of sensory block (in minute)	90 (60–135)	135 (105–165)	*P* = 0.000*
Duration of motor block (in minute)	195 (165–225)	255 (210–285)	*P* = 0.000*
Duration of effective analgesia (in minute)	220 (165–265)	320 (250–450)	*P* = 0.000*

*Mann-Whitney *U* test.

**Table 3:** Comparison of intraoperative events in patients of two groups.

	Group B (*n* = 45)	Group BM (*n* = 45)	Significance
Hypotension	28/45	31/45	NS*
Bradycardia	3/45	2/45	NS*
Nausea-vomiting	5/45	2/45	NS*
Respiratory depression	0/45	0/45	NS*
Sedation (0/1/2/3)	43/2/0/0	35/6/4/0	*P* = 0.017^+^
Incomplete block	0/45	0/45	NS

*Mantel-Haenszel chi-square.

^+^Fisher exact probability test.

**Table 4:** Comparison of postoperative complications in two groups.

	Group B (*n* = 45)	Group BM (*n* = 45)	Significance
PONV	9/45	2/45	*P* = 0.025*
Sedation (0/1/2/3)	44/1/0/0	42/2/1/0	NS
Respiratory depression	0/45	0/45	NS
Pruritus	0/45	0/45	NS

*Mantel-Haenszel chi-square.
